# Presumptive Treatment of Malaria from Formal and Informal Drug Vendors in Nigeria

**DOI:** 10.1371/journal.pone.0110361

**Published:** 2014-10-21

**Authors:** Chinwoke Isiguzo, Jennifer Anyanti, Chinazo Ujuju, Ernest Nwokolo, Anna De La Cruz, Eric Schatzkin, Sepideh Modrek, Dominic Montagu, Jenny Liu

**Affiliations:** 1 Research and Evaluation Division, Society for Family Health, Abuja, Nigeria; 2 Technical Services Division, Society for Family Health, Abuja, Nigeria; 3 Global Fund Malaria Division, Society for Family Health, Abuja, Nigeria; 4 The Global Health Group, University of California San Francisco, San Francisco, California, United States of America; 5 General Medical Disciplines, Stanford University School of Medicine, Palo Alto, California, United States of America; 6 Epidemiology and Biostatistics, University of California San Francisco, San Francisco, California, United States of America; Royal Tropical Institute, Netherlands

## Abstract

**Background:**

Despite policies that recommend parasitological testing before treatment for malaria, presumptive treatment remains widespread in Nigeria. The majority of Nigerians obtain antimalarial drugs from two types of for-profit drug vendors—formal and informal medicine shops—but little is known about the quality of malaria care services provided at these shops.

**Aims:**

This study seeks to (1) describe the profile of patients who seek treatment at different types of drug outlets, (2) document the types of drugs purchased for treating malaria, (3) assess which patients are purchasing recommended drugs, and (4) estimate the extent of malaria over-treatment.

**Methods:**

In urban, peri-urban, and rural areas in Oyo State, customers exiting proprietary and patent medicine vendor (PPMV) shops or pharmacies having purchased anti-malarial drugs were surveyed and tested with malaria rapid diagnostic test. A follow-up phone survey was conducted four days after to assess self-reported drug administration. Bivariate and multivariate regression analysis was conducted to determine the correlates of patronizing a PPMV versus pharmacy, and the likelihood of purchasing an artemisinin-combination therapy (ACT) drug.

**Results:**

Of the 457participants who sought malaria treatment in 49 enrolled outlets, nearly 92% had diagnosed their condition by themselves, a family member, or a friend. Nearly 60% pharmacy customers purchased an ACT compared to only 29% of PPMV customers, and pharmacy customers paid significantly more on average. Multivariate regression results show that patrons of PPMVs were younger, less wealthy, waited fewer days before seeking care, and were less likely to be diagnosed at a hospital, clinic, or laboratory. Only 3.9% of participants tested positive with a malaria rapid diagnostic test.

**Conclusions:**

Poorer individuals seeking care at PPMVs are more likely to receive inappropriate malaria treatment when compared to those who go to pharmacies. Increasing accessibility to reliable diagnosis should be explored to reduce malaria over-treatment.

## Introduction

Nigeria bears one of the world's highest burdens of malaria, accounting for a quarter of all cases in Africa [1]. It is estimated that over half of Nigeria's population experiences at least one episode of malaria each year, accounting for approximately 20% of all hospital admission, 30% of outpatient visits, and 10% of hospital deaths [2]. This burden of disease strains the resources of the health system as spending on malaria treatment and prevention accounts for nearly 50% of all health expenditures in Nigeria [3].

To effectively diagnose and treat malaria, the World Health Organization (WHO) currently recommends a confirmatory blood test for all suspected cases of malaria and prescription of artemisinin-based combination therapy (ACT) upon confirmation of malaria positivity [4]. ACTs are currently the most effective antimalarial treatment and are becoming more widely available in Nigeria [5]. However, many health care providers in Nigeria continue to prescribe less-effective drugs, such as chloroquine (CQ) and sulfadoxine-pyrimethamine (SP), for uncomplicated cases of malaria [6]. Despite the increased availability of malaria rapid diagnostic tests (RDTs) to facilitate point-of-care diagnosis elsewhere in sub-Saharan Africa [7]–[11], RDTs are not yet widely available in Nigeria [12] and presumptive diagnosis continues to be the most common method for determining a patient's malaria status [13].

This study aims to better characterize the practice of presumptive treatment of malaria in Nigeria and determine where interventions for malaria treatment delivery should be targeted. Nearly 60% of Nigerians seek treatment for malaria at drug shop outlets in the private healthcare sector [14]. Of these vendors, the minority is composed of licensed pharmacies, which are either owned or staffed by formally trained pharmacists, and which are mainly found in urban centers. The majority of vendors are informally trained, loosely regulated proprietary and patent medicine vendors (PPMVs), which are frequently the only source of drugs in rural areas [14]–[16]. While both types of vendors mainly operate as drug retailers, the quality of health services offered can vastly differ. PPMVs are legally permitted to only sell a number of medications over-the-counter, including antimalarial medications, but recent assessments show that they often do not stock ACTs, and have limited knowledge of malaria symptoms and recommended treatment guidelines [17]. In contrast, pharmacists are perceived to offer higher-quality malaria care services than PPMVs [18], although little empirical evidence exists to corroborate these views.

When choosing the type of facility at which to seek care, patients may prioritize convenience, availability of familiar drugs, and affordability [18]. While hospitals and clinics may provide higher quality care and testing, long wait and travel times often drive patients to accessible, nearby drug vendors. Similarly, there is little demand for confirmatory malaria microscopy testing, leading many people to bypass hospital/clinics or costly independent diagnostic laboratories [19].

Because private sector drug vendors are the source for such a large proportion of Nigeria's population seeking malaria care, it is important to understand the extent to which individuals seeking treatment for malaria are able to receive accurate diagnosis and treatment at pharmacies and PPMVs [20]. It is also important to understand what types of consumers may be at most risk for receiving poor quality services. Consequently, this study seeks to (1) describe and compare the profile of patients who seek treatment at PPMVs versus pharmacies, (2) document the types of drugs purchased for treating malaria, (3) assess which patients are purchasing recommended ACTs, and (4) estimate the extent of malaria over-treatment [21]. Implications of findings for targeting appropriate diagnostic and treatment interventions are discussed.

## Materials and Methods

### Ethical considerations

The Nigerian Health Research Ethical Review Committee (NHREC Approval Number NHREC/01/01/2007-30/08/2012) and the University of California, San Francisco's Committee for Human Research approved all study protocols. Data collectors obtained written informed consent from study participants and shop proprietors where the customers were recruited. Written consent was obtained from shop proprietors via signature and customers via signature or fingerprint for non-literate customers. The consent procedure was approved by the Nigerian Health Research Ethical Review Committee and the University of California, San Francisco's Committee for Human Research. Funding sponsors for the study did have any role in the study design, execution, or publication.

### Study area and sample selection

The study was conducted in Oyo State, located in the Southwest geopolitical zone of Nigeria, comprised of about 4.5million people (predominantly of Yoruba descent) [13]. Four local government areas (LGAs) were purposefully selected for the study to include urban, semi-urban, and rural areas: Ibadan South East (urban) andEgbeda (semi-urban) in and around the Ibadancity area, whileOgbomosho South and OgoOluwa were selected in and around the Ogbomosho town area (rural). All PPMV and pharmaceutical shops were first enumerated within the four selected LGAs and a total number of 236 PPMVs and 24 pharmaceutical shops were identified during the enumeration exercise. Interviewers used a questionnaire that captured the names, addresses, location (urban, seri-urban, rural), LGA, GPS coordinates, notable landmarks, and size of outlets (‘small’ for outlets with two shelves of medicines; ‘medium’ for outlets with three to four shelves of medicine; ‘large’ for outlets with more than five shelves).

From the complete list of shops, a total of fifty pharmacies and PPMVs were randomly selected, stratified by the size of medicinal stock (i.e. small, medium, and large). Selected shops were visited to inform shop owners of the study aims and obtain permission to recruit exiting customers into the study. Enrolled study sites were later modified to exclude 24 small PPMV drug retailers in Ibadan whose main business was not medicinal sales (thus participants were not able to be recruited) and replaced with randomly selected PPMV shops in Ibadan North East LGA. Using a standard script, 49 out of 50 selected private sector retailers (42shop in/around Ibadan, 7shops in/around Ogbomosho) agreedto havetheir shop used for participant recruitment and were enrolled into the study; only one pharmacy declined to participatefor reasons not stated. The final roster of recruitment sites consisted of 21 pharmacies and 23 PPMVs. All seven shops enrolled in Ogbomosho were PPMVs as the city did not have any pharmacies during site enumeration.

Two members of the survey team, one trained nurse and one researcher, were stationed at enrolled PPMVs and pharmacies on randomly selected days of the week (excluding Sunday) and approached customers as they exited the drug store to assess eligibility. The inclusion criteria were as follows: the participant must be a non-pregnant adult having purchased treatment for malaria for him-or herself and be willing to complete a 15-minute survey. Malaria “treatment” was defined to mean any drug purchased by the customer that s/he intended to take for their current episode of suspected malaria, which may include inappropriate drugs for malaria and not necessarily an antimalarial drug. While seeking consent, the participant was informed that if they qualified, they would be offered a RDT and would be compensated for their time with a small mobile phone credit of 200 Naira (∼US$1.20) for participating in the interview.

### Data collection

Two surveys were conducted, one at the time of enrollment and testing (i.e. baseline) and one after four days of the initial encounter via telephone call(i.e. follow-up). All data were collected concurrently. At baseline, the eligible participant was offered a RDT performed by a trained nurse at the beginning of the survey. While the RDT result was pending (about 15 minutes), a detailed survey was conducted designed to capture information on the background demographics and socioeconomic stats, symptoms experienced, and care-seeking actions taken for the current and past episodes of suspected malaria. Contact information was also collected during enrollment to facilitate later follow-up. At the end of the survey, the participant was provided with the result of his or her test.

Nurses were instructed to provide participants with standard advice according to their RDT results. If the participant tested positive for malaria, he/she was told that the positive result suggests the presence of malaria. Per ethical considerations to ensure that the participants testing positive had a quality-assured anti-malaria drug, a free course of ACTs was provided and participants were instructed to take it according to the recommended dosage protocol. If the test was negative, the participant was told that the negative result indicates the absence of malaria and that anti-malarial drugs they purchased were not needed. Regardless of the test result, all participants were referred to local clinics and hospitals where they could seek care if their condition was not malaria, or if their illness became worse. All participants were told to expect a short 5–10 minute follow up phone call in four days to check on the status of their illness and that they would be compensated with a small phone credit of 100 Naira (∼US$0.60)for taking the call.

Four days after the baseline survey, a nurse called the participants and conducted a phone survey to obtain information on the state of their health and the drugs they had used. A total of 465 adults were enrolled in the baseline survey, but eight were excluded due to survey numbering errors, and 424 participated in the follow-up phone survey—a follow-up retention rate of 92.8%. No differences in individual characteristics between attritted and retained participants were detected; detailed sample attrition are described elsewhere [22].

### Data Analysis

#### Descriptive data analysis

Descriptive analysis was used for the study to review the sample for basic socio demographic characteristics, reasons for choosing the drug shop, and drugs purchased and taken. Wealth distribution was assessed using standard principal components analysis (PCA) [23] in two ways. First, to compare the representativeness of the study sample to the overall state and national sample, weights associated with PCA component items generated from the 2010 Nigeria Malaria Indicator Survey (MIS) were applied to comparable asset indicators collected in the study sample to compute a wealth index that reflected the national wealth distribution. This index was then converted to quintile categorical indicators for comparing external sample representativeness. A second wealth index was created using only the study sample via the same PCA technique and converted to quintile indicators to obtain an even, within-sample distribution of wealth.

#### Regression Analysis

Two types of regression analyses were conducted. First, we estimated bivariate and multivariate logistic regressions to assess differences in the types of individuals that patron different types of drug shops—PPMVs versus pharmacies. The likelihood that a PPMV was chosen was predicted by the individual's age, sex, educational attainment, marital status, and wealth. In addition to basic socio demographics, employment status (i.e. full-time wage worker, part-time wage worker, self-employed, and unemployed) was included as shop owners indicated that customers tend to stop at drug shops on their way to and from work. Self-reported symptoms felt for the current illness episode, the number of days waited before seeking care, and where the recognition of the illness as malaria came from (i.e. myself/relative/friend, a hospital/clinic/diagnostic laboratory, or at a drug retailer) were also included because these factors may influence the choice of drug shop type based on perceived severity, need for drug administration, or recommendations by diagnosticians. Second, the likelihood of buying an ACT was predicted using logistic regression analysis. In addition to individual characteristics described above, the type of shop (i.e. PPMV vs. pharmacy) was included as a risk factor for receiving the recommended first-line malaria drug. In both analyses, only statistically significant explanatory variables at the 5% level in bivariate analysis were included in the multivariate model. To account for autocorrelation between individuals recruited at the same shop, standard errors were clustered at the shop level. Odds ratios are reported.

## Findings

### Sample characteristics

Of the 457participants who sought malaria treatment from the 49enrolled shops, 71.1% (n = 325) were recruited in Ibadan, 55.6% (n = 254) were under the age of 40 (median  = 37; range: 18–82), 50.8% were male (n = 232), and 68.1% (n = 311) were married. Only 22.5% (n = 103) had primary education or less; 39.8% (n = 182) completed secondary education and 37.6% (n = 172) had some tertiary level education. Among those interviewed, 31.3% (n = 143) were employed either on a full-time or part-time basis; 53.6% were self-employed (n = 245) and 15.1% were unemployed (n = 69). Participants reported feeling a variety of symptoms during their current episode of illness. Fever was most commonly reported (74.6%, n = 341), followed by body aches, chills, or convulsions (57.5%, n = 263), feeling weak, fatigued, or having little appetite (55.8%, n = 255). Nearly half of participants waited one day (18.7%, n = 79) or less (28.7%; n = 122) before seeking care; 34.3% (n = 146) waited three days or more. Nearly 92% (n = 423) of the participants reported that they had diagnosed the episode of malaria by themselves, a family member, or a friend. See Table 1 for a summary of the sample characteristics.

**Table 1:** Customer demographic and socioeconomic variables (N = 457).

Variable		N	%
Site	Ogbomosho	132	28.9
	Ibadan	325	71.1
Age of respondents	18–29	127	27.8
	30–39	127	27.8
	40–49	100	21.9
	50+	103	22.5
Sex	Male	232	50.8
	Female	227	49.2
Education	No education	130	28.4
	Primary education	13	2.8
	Secondary education	245	53.6
	Higher education	69	15.1
Marital status	Not married	146	31.9
	Married	311	68.1
Employment status	Employed full time	130	28.4
	Employed part time	13	2.8
	Self-employed	245	53.6
	Unemployed	69	15.1
Wealth quintile^1^	Poorest	90	19.7
	Second	92	20.1
	Third	93	20.4
	Fourth	90	19.7
	Richest	92	20.1
Symptoms reported			
Fever, headache, dizziness	Yes	341	74.6
	No	116	25.4
Body aches, chills, convulsions	Yes	263	57.5
	No	194	42.5
Weak, fatigue, no appetite	Yes	255	55.8
	No	202	44.2
Bitter taste in the mouth	Yes	62	13.6
	No	395	86.4
Congestion, shallow breathing	Yes	58	12.7
	No	399	87.3
Vomiting, diarrhea	Yes	54	11.8
	No	403	88.2
Other: blisters, dark urine, yellow eyes	Yes	63	13.8
	No	394	86.2
Number of days waited before seeking care^2^	<1 day	122	28.7
	1 day	79	18.7
	2 days	77	18.2
	3–5 days	104	24.5
	6 days or more	42	9.8
Source of diagnosis	Self-diagnosis	418	91.5
	Hospital/clinic/lab	20	4.4
	Pharmacy/PPMV	19	4.2

1 Result of within-sample principle components analysis.

2 N = 424.

**Figure 1:**
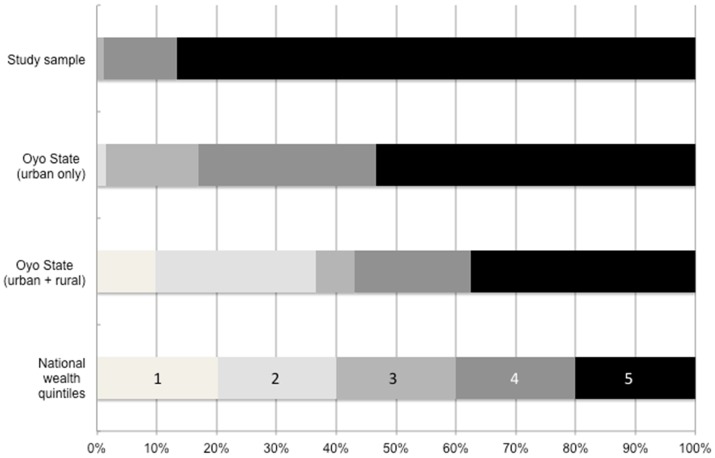
Wealth distribution of enrolled participants versus state and national populations. When comparing the wealth distribution between sampled individuals to that of the state and national populations captured by the 2010 MIS, those in the wealthiest quintile are disproportionately represented in the study sample. Based on the composite asset ownership measure, no individuals in the study sample were from the lowest two wealth quintiles of nation. Although the population of Oyo State, and particularly in urban areas, is also comprised of households that are much wealthier than the nation as a whole, the study sample's wealth composition is even more concentrated among the wealthiest. Source: 2010 Nigeria Malaria Indicators Survey.

When comparing the wealth distribution between sampled individuals to that of the state and national populations captured by the 2010 MIS, those in the wealthiest quintile are disproportionately represented in the study sample. Based on the composite asset ownership measure, no individuals in the study sample were from the lowest two wealth quintiles of nation as seen in Figure 1. Although the population of Oyo State, and particularly in urban areas, is also comprised of households that are much wealthier than the nation as a whole, the study sample's wealth composition is even more concentrated among the wealthiest.

### Types of medicines purchased for malaria treatment

Table 2 summarizes the types of drugs purchased at PPMVs and pharmacies for the current episode of suspected malaria among participants who agreed to have their drugs examined by the study nurse (n = 423). A significantly higher percentage of the patrons of pharmacies (57.4%, n = 132/230) purchased an ACT compared to only 28.5% (n = 55/193) of PPMV patrons (p<0.01). Of non-ACT antimalarials purchased, significantly more customers at PPMVs (47.7%; n = 92/230) purchased SP than customers of pharmacies (28.7%, n = 66/193; p<0.05). A higher percentage of PPMV customers also bought a non-malaria drug (70.1%, n = 136/194) compared to pharmacy customers (54.7%, n = 129/236). Significantly fewer analgesics (76.0%, n = 98/230), but more vitamins/supplements (85.3%, n = 110/230) and antibiotics (20.9%, n = 27/230) were purchased at pharmacies than at PPMVs (respectively: 92.6%, n = 126/193; 60.3%, n = 82/193; 5.9%, n = 8/193). More patrons of pharmacies bought only an antimalarial (45.3%, n = 107/230) compared to those at PPMVs (29.9%, n = 58/193). However, pharmacy customers paid significantly more for all of their drugs than those purchasing at PPMVs on average.

**Table 2:** Drugs purchased to treat malaria.

	Pharmacies		PPMVs		Total		
	n	%	n	%	n	%	P-value
Type of anti-malarial drug^1^							
ACT	132	57.4	55	28.5	187	44.2	0.003
SP	66	28.7	92	47.7	158	37.4	0.022
CQ	46	20.0	33	17.1	79	18.7	0.545
Other	21	9.1	16	8.3	37	8.7	0.830
Purchased non-malaria drug							
Yes	129	54.7	136	70.1	265	61.6	0.068
No	107	45.3	58	29.9	165	38.4	
Type of non-malaria drugs							
Analgesic	98	76.0	126	92.6	224	84.5	0.003
Vitamin/supplement	110	85.3	82	60.3	192	72.5	0.007
Antibiotic	27	20.9	8	5.9	35	13.2	0.019
Other	16	12.4	11	8.1	27	10.2	0.251
Purchase combinations							
Anti-malarial only	107	45.3	58	29.9	167	38.6	0.056
Non-malaria drug only	6	1.3	1	0.3	7	0.8	
Both anti-malarial and non-malaria drug	123	26.2	135	34.9	259	30.1	
	n	median	n	median	n	median	P-value
Total amount paid (median)	234	445	193	140	427	240	0.000

1 Pharmacies N = 230; PPMVs N = 193; Total N = 423. Not all participants purchased an anti-malarial drug.

### Correlates of seeking care at a PPMV versus a pharmacy

Results of logistics regressions predicting the likelihood of going to a PPMV versus a pharmacy for malaria treatment are summarized in Table 3. In bivariate analyses, customers going to different shop types were significantly different in terms of their age, educational attainment, employment status, and wealth. Significant differences were also registered for a number of symptoms (i.e. fever, headache, or dizziness; feeling weak, fatigues, or no appetite; having congestion or shallow breathing; and other symptoms including blusters, dark urine, and yellow eyes), days waited before seeking care, and the source of diagnosis. In multivariate analyses, older individuals are about half as likely to patron a PPMV (age 30–39: OR = 0.416, 95% CI: 0.230–0.752; age 50+: OR = 0.461, 95% CI: 0.229–0.929) than those under age 30. A strong wealth gradient emerges with the individuals in progressively richer wealth quintiles increasingly less likely to go to a PPMV compared to those in the poorest quintile. Those reporting other types of symptoms (i.e. blisters, dark urine, yellow eyes) were more than three times as likely to go to a PPMV (OR = 3.138, 95% CI: 1.381–7.128) while those reporting fever, headache, or dizziness (OR = 2.589, 95% CI: 1.501–4.465) and weakness, fatigue, and lack of appetite (OR = 1.951, 95% CI: 1.043–3.649) were about twice as likely to go to a PPMV. When individuals waited one day before seeking care, they were twice as likely to go to a PPMV (OR = 2.070, 95% CI: 1.256–3.411) compared to those who sought treatment the same day; the likelihood of PPMV patronage progressively declined as the number of days waited increased, but these were not statistically significant. Diagnosis coming from a hospital, clinic, or laboratory was associated with a large and significantly lower likelihood of going to a PPMV (OR = 0.022, 95% CI: 0.137–1.311). Education and employment status were no longer statistically significant after adjusting for all confounders.

**Table 3:** Logistic regression of the likelihood of buying drugs from a PPMV (versus a pharmacy).

		Pharmacy		PPMV			Bivariate			Multivariate		
		n = 245	%	n = 212	%	P-value	OR^1^	95% CI	P-val	OR^1^	95% CI	P-val
Age of respondents	18–29 (reference)	52	21.2	74	34.9	0.005	1.000			1.000		
	30–39	78	31.8	49	23.1		0.441***	0.275–0.709	0.001	0.416***	0.230–0.752	0.004
	40–49	56	22.9	44	20.8		0.552	0.268–1.139	0.108	0.564	0.255–1.249	0.158
	50+	59	24.1	45	21.2		0.524**	0.292–0.939	0.030	0.461**	0.229–0.929	0.030
Sex	Male	125	51.0	106	50.0	0.893	0.969	0.615–1.528	0.893			
	Female (reference)	120	49.0	106	50.0		1.000					
Education	No education (reference)	10	4.1	28	13.2	0.012	1.000			1.000		
	Primary education	24	9.8	42	19.8		0.610	0.247–1.504	0.283	0.984	0.319–3.033	0.977
	Secondary education	102	41.6	79	37.3		0.277**	0.102–0.749	0.012	0.470	0.157–1.407	0.177
	Higher education	109	44.5	63	29.7		0.206***	0.064–0.667	0.008	0.607	0.136–2.712	0.514
Marital status	Not married (reference)	66	26.9	80	37.7	0.089	1.000					
	Married	179	73.1	132	62.3		0.616*	0.352–1.077	0.089			
Employment status	Employed full time (reference)	83	33.9	47	22.3	0.012	1.000			1.000		
	Employed part time	8	3.3	5	2.4		1.104	0.240–5.078	0.899	0.463	0.085–2.520	0.373
	Self-employed	111	45.3	134	63.0		2.116***	1.205–3.717	0.009	1.337	0.627–2.849	0.452
	Unemployed	43	17.6	26	12.3		1.068	0.581–1.962	0.833	0.761	0.299–1.940	0.567
Wealth quintile	Poorest (reference)	21	8.6	68	32.1	0.000	1.000			1.000		
	Second	38	15.5	54	25.5		0.439***	0.235–0.819	0.010	0.430**	0.205–0.900	0.025
	Third	53	21.6	40	18.9		0.233***	0.126–0.431	0.000	0.205***	0.087–0.487	0.000
	Fourth	59	24.1	32	15.1		0.162***	0.072–0.368	0.000	0.152***	0.056–0.413	0.000
	Richest	74	30.2	18	8.5		0.0751***	0.023–0.249	0.000	0.075***	0.018–0.318	0.000
Symptoms reported												
Fever, headache, dizziness	Yes	168	68.6	172	81.1	0.001	2.021***	1.317–3.101	0.001	2.589***	1.501–4.465	0.000
	No (reference)	77	31.4	40	18.9		1.000			1.000		
Body aches, chills, convulsions	Yes	130	53.1	133	62.7	0.096	1.508*	0.929–2.448	0.096			
	No (reference)	115	46.9	79	37.3		1.000					
Weak, fatigue, no appetite	Yes	120	49.0	134	63.2	0.011	1.813**	1.144–2.872	0.011	1.951**	1.043–3.649	0.036
	No (reference)	125	51.0	78	36.8		1.000			1.000		
Bitter taste in the mouth	Yes	29	11.8	33	15.6	0.241	1.381	0.806–2.367	0.240			
	No (reference)	216	88.2	179	84.4		1.000					
Congestion, shallow breathing	Yes	38	15.5	20	9.4	0.016	0.570**	0.361–0.902	0.016	0.619*	0.372–1.030	0.065
	No (reference)	207	84.5	192	90.6		1.000			1.000		
Vomiting, diarrhea	Yes	30	12.2	24	11.3	0.752	0.920	0.548–1.545	0.752			
	No (reference)	215	87.8	188	88.7		1.000					
Other: blisters, dark urine, yellow eyes	Yes	15	6.1	48	22.6	0.000	4.515***	2.345–8.696	0.000	3.138***	1.381–7.128	0.0063
	No (reference)	230	93.9	164	77.4		1.000			1.000		
Number of days waited before seeking care^2^	<1 day (reference)	56	25.9	65	31.7	0.016	1.000			1.000		
	1 day	30	13.9	49	23.9		1.378	0.854–2.225	0.189	2.070***	1.256–3.411	0.004
	2 days	37	17.1	40	19.5		0.931	0.510–1.700	0.817	1.624	0.890–2.962	0.114
	3–5 days	66	30.6	37	18.0		0.483*	0.220–1.058	0.069	0.659	0.289–1.504	0.322
	6 days or more	27	12.5	14	6.8		0.447*	0.181–1.100	0.080	0.431	0.137–1.355	0.150
Source of diagnosis	Myself/family/friend (reference)	213	86.9	205	96.7	0.011	1.000			1.000		
	Hospital/clinic/lab	19	7.8	1	0.5		0.055***	0.008–0.393	0.004	0.022**	0.001–0.412	0.011
	Pharmacy/PPMV	13	5.3	6	2.8		0.482	0.149–1.557	0.222	0.424	0.137–1.311	0.136
Observations							457			420		

1 Odds ratios reported.

2 Pharmacy (n = 212), PPMV (n = 205), Total (n = 417).

Standard errors are clustered at the shop level;

***p<0.01,

**p<0.05,

*p<0.1.

**Figure 2:**
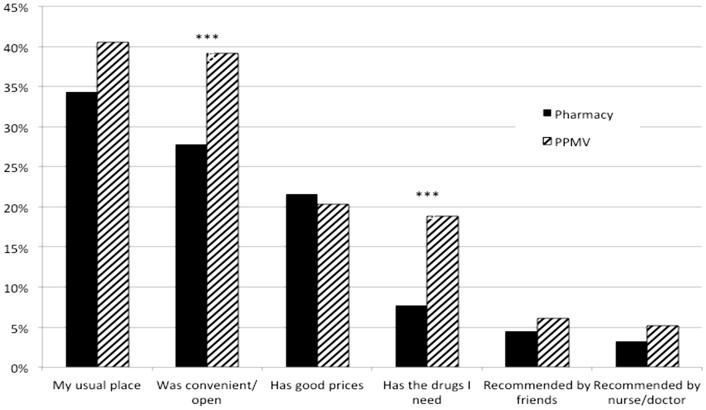
Reasons for choosing a drug shop (N = 457). When asked for reasons why they chose the particular drug shop, most respondents stated reasons of habit and convenience (see Figure 2). A significantly higher percentage of participants at PPMVs said that the shop was convenient and had the drugs that s/he needed. In similar percentages, both types of outlets were cited for their prices. Note: *** p<0.01, ** p<0.05, * p<0.1.

When asked for reasons why they chose the particular drug shop, most respondents stated reasons of habit and convenience (see Figure 2). A significantly higher percentage of participants at PPMVs said that the shop was convenient and had the drugs that s/he needed. In similar percentages, both types of outlets were cited for their prices.

### Predictors of purchasing an ACT

The logistic regression results predicting the likelihood of purchasing an ACT over other types of antimalarial drugs are listed in Table 4. In bivariate analyses, shop type, wealth, and the source of diagnoses were the only factors that were significantly associated with the likelihood of buying an ACT. In adjusted regressions, customers who went to PPMVs were significantly less likely to buy an ACT (OR = 0.371, 95% CI: 0.168-0.821). While having a diagnosis from a hospital, clinic, or laboratory was associated with increased likelihood of ACT purchase, this was only marginally significant at the 10% level. Differences in wealth were no longer significant in the multivariate specification, although a gradient was still observed.

**Table 4:** Logistic regression of the likelihood of buying an ACT (versus other anti-malarial drugs).

		ACT		Other anti-malarial			Bivariate			Multivariate		
		n = 233	%	n = 184	%	P-value	OR^1^	95% CI	P-val	OR^1^	95% CI	P-val
Type of shop^1^	PPMV	136	58.4	136	29.3	0.002	0.296***	0.135–0.650	0.002	0.371**	0.168–0.821	0.015
	Pharmacy	97	41.6	97	70.7		1.000			1.000		
Age of respondents	18–29 (reference)	70	30.0	70	22.3	0.541	1.000					
	30–39	64	27.5	64	29.3		1.441	0.800–2.595	0.224			
	40–49	52	22.3	52	23.9		1.445	0.775–2.694	0.247			
	50+	47	20.2	47	24.5		1.635	0.767–3.485	0.203			
Sex	Male	125	53.6	125	48.4	0.398	0.809	0.496–1.322	0.398			
	Female (reference)	108	46.4	108	51.6		1.000					
Education	No education (reference)	18	7.7	18	6.5	0.182	1.000					
	Primary education	36	15.5	36	9.8		0.750	0.300–1.873	0.538			
	Secondary education	96	41.2	96	39.1		1.125	0.513–2.466	0.769			
	Higher education	83	35.6	83	44.6		1.482	0.647–3.395	0.352			
Marital status	Not married (reference)	76	32.6	76	29.9	0.654	1.000					
	Married	157	67.4	157	70.1		1.135	0.652–1.978	0.654			
Employment status	Employed full time (reference)	59	25.3	59	34.2	0.105	1.000					
	Employed part time	6	2.6	6	2.7		0.780	0.174–3.499	0.746			
	Self-employed	136	58.4	136	46.2		0.585**	0.368–0.930	0.023			
	Unemployed	32	13.7	32	16.8		0.907	0.541–1.522	0.712			
Wealth quintile	Poorest (reference)	50	21.5	50	14.7	0.008	1.000			1.000		
	Second	59	25.3	59	14.1		0.816	0.454–1.466	0.497	0.652	0.357–1.192	0.165
	Third	48	20.6	48	17.4		1.235	0.610–2.500	0.558	0.875	0.412–1.861	0.730
	Fourth	44	18.9	44	23.9		1.852	0.870–3.943	0.110	1.234	0.615–2.477	0.554
	Richest	32	13.7	32	29.9		3.183***	1.499–6.758	0.003	1.931	0.880–4.236	0.101
Symptoms reported												
Fever, headache, dizziness	Yes	171	73.4	171	73.4	0.996	0.999	0.635–1.571	0.996			
	No (reference)	62	26.6	62	26.6		1.000					
Body aches, chills, convulsions	Yes	136	58.4	136	57.1	0.818	0.948	0.601–1.494	0.818			
	No (reference)	97	41.6	97	42.9		1.000					
Weak, fatigue, no appetite	Yes	138	59.2	138	54.3	0.387	0.820	0.522–1.287	0.387			
	No (reference)	95	40.8	95	45.7		1.000					
Bitter taste in the mouth	Yes	31	13.3	31	14.7	0.654	1.121	0.681–1.844	0.654			
	No (reference)	202	86.7	202	85.3		1.000					
Congestion, shallow breathing	Yes	24	10.3	24	16.3	0.110	1.696	0.887–3.244	0.110			
	No (reference)	209	89.7	209	83.7		1.000					
Vomiting, diarrhea	Yes	26	11.2	26	10.9	0.916	0.971	0.561–1.679	0.916			
	No (reference)	207	88.8	207	89.1		1.000					
Other: blisters, dark urine, yellow eyes	Yes	33	14.2	33	11.4	0.448	0.781	0.412–1.480	0.448			
	No (reference)	200	85.8	200	88.6		1.000					
Number of days waited before seeking care^1^	<1 day (reference)	68	30.8	68	24.8	0.140	1.000					
	1 day	46	20.8	46	15.5		0.924	0.455–1.875	0.827			
	2 days	42	19.0	42	18.6		1.214	0.691–2.135	0.500			
	3–5 days	45	20.4	45	29.2		1.776**	1.038–3.039	0.0362			
	6 days or more	20	9.0	20	11.8		1.615	0.798–3.269	0.183			
Source of diagnosis	Myself/family/friend (reference)	219	94.0	219	89.1	0.019	1.000			1.000		
	Hospital/clinic/lab	4	1.7	4	7.1		4.340**	1.323–14.24	0.0155	3.124*	0.987–9.894	0.053
	Pharmacy/PPMV	10	4.3	10	3.8		0.935	0.345–2.530	0.894	0.758	0.301–1.909	0.557
Observations							417			417		

1 Odds ratios reported.

2 Pharmacy (n = 221), PPMV (n = 161), Total (n = 382).

Standard errors are clustered at the shop level;

***p<0.01,

**p<0.05,

*p<0.1.

### RDT results and self-reported drug administration

Of the 457 enrolled participants, 3.9% (n = 18) were RDT-positive as seen in Table 5. During the phone follow-up survey, 97.9% (n = 415/424) of those reached reported that they felt better than the day they were enrolled into the study and 5.9% (n = 25/422) had sought additional care. For those who were RDT-positive (and for whom drug information is available), 68.8% (n = 11/16) reported taking an ACT; none took a non-ACT anti-malarial and all took some type of non-anti-malarial drug that they had also purchased (n = 12). Among RDT-negative participants, 28.9% still used some form of anti-malarial medication: 9.7% (n = 39/402) took an ACT and 19.2% (n = 77/402) took a non-ACT anti-malarial. For those who also purchased a non-anti-malarial, 76.5% (n = 189/247) took these drugs. When asked which places could be trusted to provide RDTs, 77.4% (n = 328/425) indicated hospitals or clinics and 17.5% (n = 74) named a diagnostic laboratory; only 4.2% stated pharmacies, 1.2% (n = 5) named PPMVs, and 3.5% (n = 15) indicated a family or friend could be trusted.

**Table 5:** RDT result and self-reported drug administration.

		n	N	%
RDT result	Positive	18	457	3.9
	Negative	439	457	96.1
Generally feeling better since baseline	Yes	415	424	97.9
	No	9	424	2.1
Drugs taken				
RDT-positive				
ACT	Yes	11	16	68.8
	No	5	16	31.3
Non-ACT anti-malarial	Yes	0	16	0
	No	0	16	0
Non-anti-malarial	Yes	12	12	100
	No	0	12	0
RDT-negative				
ACT	Yes	39	402	9.7
	No	363	402	90.3
Non-ACT anti-malarial	Yes	77	402	19.2
	No	325	402	80.8
Non-anti-malarial	Yes	189	247	76.5
	No	58	247	23.5
Sought additional care	Yes	25	422	5.9
	No	397	422	94.1
Places trusted to provide RDTs				
Hospital/clinic	Yes	328	424	77.4
	No	96	424	22.6
Diagnostic lab	Yes	74	424	17.5
	No	350	424	82.5
Pharmacy	Yes	18	424	4.2
	No	406	424	95.8
PPMV	Yes	5	424	1.2
	No	419	424	98.8
Community health worker	Yes	23	424	5.4
	No	401	424	94.6
Traditional healer	Yes	4	424	0.9
	No	420	424	99.1
Family/friend	Yes	15	424	3.5
	No	409	424	96.5
Felt well at follow up and RDT positive				
Took ACTs	Yes	9	16	56.3
	No	7	16	43.7
Took non- ACT anti-malarial	Yes	7	16	43.7
	No	9	16	56.7
Non- antimalarial	Yes	0	16	0
	No	16	16	100
Felt well at follow up and RDT negative				
Took ACTs	Yes	206	389	53.0
	No	183	389	47.0
Took non- ACT anti-malarial	Yes	181	389	46.5
	No	208	389	53.5
Non- antimalarial	Yes	2	389	0.5
	No	387	389	99.5

## Discussion

Like elsewhere in sub-Saharan Africa [17], [24]–[26], medicine retailers in Nigeria are an important source of treatment for uncomplicated malaria even though the quality of care and knowledge among these providers is poorer compared to other types of health professionals. This study sought to better characterize the practice of presumptive treatment at drug outlets in Nigeria. Recruited as they exited drug shops, nearly all study participants reported that they had self-diagnosed their condition and chose to patron the particular shop because it was either their usual place to buy drugs or was convenient. Individuals who went to PPMVs were typically younger, poorer, waited fewer days before seeking care, and had not gone to a hospital/clinic for diagnosis. These results suggest that relatively poorer populations, and potentially less-educated, are disproportionately serviced by PPMVs, potentially motivated by a variety of factors, including proximity and accessibility [27], [28] and cost [29], [30].

In this study, even though patrons of PPMVs spent less on their drug purchases on average, they were also more likely to purchase sub-standard, non-ACT anti-malarials. Going to a PPMV was the largest risk factor for not buying an ACT, indicating that PPMVs continue to sell non-recommended drugs for malaria. Studies show that receiving ACTs is highly associated with consumer demand [6], and that PPMVs in particular tend to sell what customers demand and avoid referring patients for confirmatory blood tests because they fear losing customers due to added inconvenience or cost [18]. Thus, consumer preferences for presumptive treatment and non-recommended drugs may drive individuals to patron PPMVs rather than pharmacies, even both types of outlets are generally perceived to provider lower quality services than hospitals or clinics [31]. Profit motives may further constrain proper dispensing behavior due to strong consumer demand for substandard drugs [26].

In addition to fever, many participants attributed a wide variety of symptoms to malaria, and those going to PPMVs were especially likely to name symptoms unrelated to malaria. Further, only 3.9% of sick adults seeking care for malaria were found to be positive for malaria using an RDT. This corroborates qualitative evidence that malaria is identified as the illness for a large swath of conditions in Nigeria [18], and that malaria is over-diagnosed and over-treated, similar to other urban areas of Nigeria [32] and elsewhere in sub-Saharan malaria-endemic countries [33]–[36]. There is also an entrenched perception that malaria is rampant and many people have been regaled with the need to treat malaria promptly by public health messages in the past aimed at increasing awareness. This highlights the importance of malaria behavior change messages that inform people that not all fever cases can be attributed to malaria and the need for individuals to seek out malaria diagnosis.

Although the study did not have a comparison control group, it is assumed that most sick individuals who purchased malaria drugs in this study were intending to take their purchased drugs to presumptively treatment him/herself. Over 44% of participants bought ACTs and the median amount spent on purchasing anti-malarial drugs was 240 Naira (∼US$1.50). This level of overtreatment suggests that large quantities of ACTs may be wasted if reliable testing is not first carried out. It is therefore imperative to train all health workers as well as the populace on malaria symptoms and the need for a diagnostic test has become vital in health communications and education. Since PPMVs and pharmacies serve the majority of the population seeking treatment for malaria, standard diagnostic testing prior to treatment should be considered as part of a concerted strategy for malaria control. Some countries, including Tanzania, Senegal, and Zambia have successfully implemented RDTs in the public health sector and provider acceptability has improved over time, resulting in sizable cost-savings [7]–[10]. Further, this is the first study to assess patient adherence to test results (rather than provider prescription behavior) showing that simply providing diagnostic information to sick individuals can result in high rates of appropriate treatment behavior [22].

In addition, this also underscores the need to provide alternative means of management of non-malaria febrile illness. There is currently not a good understanding of the etiology of non-malaria febrile illnesses and only a handful of studies have documented the variety of causes of pediatric illnesses in select countries [33], [37]. Such information is vital for developing more comprehensive treatment guidelines for childhood illnesses that are country-relevant and should be the focus of future studies.

### Limitations of the study

Populations in Southwest Nigeria are likely to be from higher socioeconomic status than populations in other areas of the country and consequently likely to be healthier overall [14]. A sample of adults as carried out in the study may not necessarily be representative of children under five who are yet to develop immunity to malaria. Further studies will be needed to look at the incidence of malaria in this group as well as in other parts of Nigeria to determine if this picture is consistent nationwide. Characteristics of the shop and its workers may also be important determinants of shop patronage and buying drugs which the current study was not able to capture, but which future studies will aim to include in the assessment of care-seeking behavior for malaria.

The study could not look at counterfeit or substandard drugs purchased at the medicine shops. However, quality-assured ACTs were given free of charge in order to address the risk of participants taking substandard drugs. Although shop workers did not participate in the recruitment or screening of customer participants, they may have altered their sales or prescriptive behavior in ways that we cannot account for.
